# A Guide to Nature Immersion: Psychological and Physiological Benefits

**DOI:** 10.3390/ijerph17165989

**Published:** 2020-08-18

**Authors:** Pei Yi Lim, Denise Dillon, Peter K. H. Chew

**Affiliations:** Psychology Department, James Cook University, Singapore 387380, Singapore; peiyi.lim@my.jcu.edu.au (P.Y.L.); peter.chew@jcu.edu.au (P.K.H.C.)

**Keywords:** forest therapy, nature immersion, nature connectedness, environmental identity, psychological benefits of nature

## Abstract

Nature exposure has been renowned for its positive physiological and psychological benefits. Recent years have seen a rise in nature immersion programs that make use of Guided Forest Therapy walks in a standard sequence of sensory awareness activities to expose participants to natural environments in a safe but effective manner. The study aimed to compare the efficacy of guided versus unguided nature immersion, upon three dependent variables of mood, nature connectedness and heartrate. 51 participants were assigned to either guided or unguided nature immersion. Nature connectedness (Connectedness to Nature Scale, CNS), Environmental Identity Scale, EID short form) and mood (Positive and Negative Affect Scale, PANAS) were assessed before and after nature immersion, while heart rate was tracked continuously by a wristwatch heart rate tracker throughout the 2-h experience. Demographics and general health practice (GHP) information were also collected. A mixed model ANOVA revealed that nature connectedness and mood (but not heart rate) improved post-immersion for all participants. Comparing the guided/unguided conditions, there were no significant differences in the change in nature connectedness, mood or heart rate. Comparing within the five segments within the standard sequence in the guided condition, the third and fifth segments revealed a significantly lower heart rate compared to the baseline heart rate.

## 1. Introduction

Worldwide, countries are gradually losing their ecosystems, as natural landscapes are being replaced by human-built features and infrastructures [1,2]. According to the World Health Organisation [3], more than half of the world’s population has lived in an urban environment since 2014, and this is expected to increase to 65% by 2030. Along with the progression of urbanization, there has been an increasing amount of urban land and a decrease in forested habitats [4,5]. In Singapore, a highly-urbanised city-state, despite aims aligned with greening policies to develop and retain its status as a “garden city” or “city in a garden”, progression towards urbanisation since the 1950s has led to a loss of 90% of its original forest [6,7,8]. This reduction in access to natural environments has gradually influenced how individuals react to and interact with nature as well as increasing disengagement [9,10,11,12].

Such disengagement from nature often propels individuals to spend a greater amount of time in built environments and engaging in technology-based activities, such as playing video games or other digital entertainment, instead of interacting with wildlife in what now requires clarification as to the “real world” [12]. It is no surprise that individuals are gradually drawn away from natural environment connections. American youths between ages 8–18 years have been found to spend an estimated seven hours each day engaged with media and technology [13]. This is a 90-min increase compared to a similar study conducted in 1999 [1]. In a separate study in 1998, Evans and McCoy [14,15] revealed that individuals at that time spent approximately 90% of their lives in buildings. Terms such as “extinction of experience” and “nature deficit disorder” have been coined to illustrate the lack of or reduced direct contact with the natural environment [2,15,16]. Granted, the use of the term “disorder” is itself contested by some parties who argue that it can be taken to promote a medicalized notion whereby the disorder is within children themselves.

### 1.1. Nature Exposure Research

While the term “nature” appears to be simple and known to all, variations in understanding of this complex term can be observed between individuals, especially among the urban population [16]). For this paper, we define nature as “an organic environment where the majority of ecosystem processes are present (e.g., birth, death, reproduction, the relationship between species)” [17] (p. 46). Nature-related research typically involves three types of contact with nature: viewing nature, being in nature and active engagement in nature-related activities (e.g., meditation in forests, gardening) [18,19]. A wealth of research has demonstrated the benefits of nature exposure on one’s physical, psychological and physiological well-being [20,21,22].

For instance, Ulrich [23] found that patients who stayed in a hospital room with a view of natural landscapes received fewer negative evaluations by the nurses, had shorter periods of stay after surgery and took fewer potent analgesics than matched participants who stayed in a room with a window view of a building wall. This suggests that there are restorative effects and physical health improvements to be gained from simple exposure to nature. A large-scale observational study in England assessed a population of over 40 million residents below retirement age and revealed an association between green-space exposure (of at least 5 m^2^) near a residential area and all-cause mortality [24].

Other research has also demonstrated that nature exposure can help to increase levels of relaxation, through physiological indices such as reduced heart rate and blood pressure [25]. Laumann, Garling and Stormark [26] recruited a sample of 28 female undergraduate students who viewed a video of either nature or urban environments. An electrocardiogram was used to assess the heart rate. The baseline heart rate, as well as heart rate and cardiac inter-beat interval (IBI) averages, were reported. It was found that participants who viewed the nature video showed a lower average heart rate compared to their baseline heart rate, while participants who watched the urban video showed no changes in their heart rate levels. In another study, Yin et al. [27] exposed participants to an indoor biophilic-designed office room with potted plants and windows with nature view versus a classroom without any green elements. Biophilic design refers to the incorporation of natural elements into a built environment.

Participants were exposed to their room environment virtually first, before physical exposure. The biophilic office room was found to induce a calming effect observed in a decrease in blood pressure and skin conductance as compared to the classroom condition. The physiological responses were similar when participants physically observed the indoor biophilic-designed room, versus when they viewed the room through a virtual reality exposure.

Besides physical health improvements, nature exposure can bring about positive influence upon psychological constructs such as boredom, friendliness, wellbeing and liveliness [28]. However, across more than one hundred studies on nature/wildlife exposure, stress mitigation has been shown to be one of the most consistent and important psychological benefits [29,30,31].

Besides improvements to physical and psychological well-being, exposure to natural environments has been shown to bring about positive impacts on cognitive functioning [20,32]. For instance, a meta-analysis of 31 studies conducted by Ohly et al. [33] revealed how nature exposure can improve individuals’ scores on various cognitive measures.

While cognitive restoration and physiological well-being are the prominent and renowned benefits of nature exposure, there is one important construct that is often overlooked in environmental psychology research studies—that is, the human-nature relationship; also known as connectedness to nature (CN). Various environmental psychologists have different interpretations of the term. For some, CN refers to the emotional bond between human and nature; the extent to which individuals perceive themselves to be part of the natural environment and their relationship with it [34,35]. Meanwhile, Clayton [36] emphasized the importance of understanding CN through the concept of environmental identity; which examines how much an individual perceives nature to be part of one’s self-perception. Lastly, others focused on the cognitive, behavioural and experiential aspects of an individual’s connection with nature [37,38]. Despite the different understandings of CN, all these concepts purportedly help explain the human-nature relationship.

Literature has generally shown that nature exposure leads to improvement in nature connectedness among participants. Students who went through environmental education have been found to develop increased levels of nature connectedness [39,40]. Individuals who spent more time in nature tended to have higher scores on nature connectedness [41,42,43]. Further, the level of nature connectedness has been found to mediate the effects of nature exposure [44].

### 1.2. Theoretical Groundings

Three major theories have been commonly cited to explain the beneficial effects of nature exposure. First, the Kaplans’ attention restoration theory (ART) explains the cognitive gains from nature exposure [45]. According to the theory, built environments are rich with constantly-moving stimuli and objects, and the prolonged effort required to direct attention and prevent distractions through inhibition can easily lead to fatigue [20]. Meanwhile, nature exposure allows individuals to be away from daily stressors, to be exposed to vast spaces and contexts, to partake in activities that are aligned with one’s intrinsic motivation, and to experience stimuli that are “softly fascinating” [33]. The immersion experience in nature stimulates the five sensory systems which increase activities in one’s parasympathetic nervous system and leads to heightened awareness, eventually resulting in a state of relaxation [45]. Thus, nature exposure provides individuals with opportunities for restorative experiences and aids one in the recovery of attentional fatigue [46].

Second, Ulrich’s stress reduction theory suggests that exposure to unthreatening natural environments can be effective in reducing stress [47]. According to Ulrich [48], natural environments contain qualities such as water, richness, vegetation, moderate depth and complexity and focal points that were essential for human survival hundreds or thousands of years ago. Given the crucial role that nature plays from the evolutionary perspective, Ulrich et al. [47] proposed that engagement in such settings should produce an affective reaction, moderating individuals’ stress levels and physiological responses.

Third, through his biophilia hypothesis, Wilson [49] proposed that humans have an innate tendency to interact positively with nature. According to the biophilia hypothesis, since the early ages, human beings have been living in forested environments, and our brains have been wired to react and adapt well to natural settings [50]. Accordingly, natural settings are not only a means of natural resources for human gains but are also a vital aspect of human cognitive, emotional, spiritual and aesthetic growth. Contact with natural environments can help to reduce stress and enhance one’s physical and mental health [51]. Meanwhile, a lack of nature exposure can cause one to suffer psychologically [52]. Thus, when individuals engage in a non-threatening natural environment, the setting will naturally draw out strong positive responses among individuals, as observed in the research evidence of benefits from nature exposure [28,48,53].

Of course, others also highlight the fact that human animals have evolved along with environments and as such are ourselves part of nature [54].

### 1.3. Specific Nature Exposure Practices

With the vast support for the benefits of nature exposure, Japan’s Ministry of Agriculture, Forestry and Fisheries introduced the practice of shinrin-yoku in 1982, as a form of preventive healthcare [55]. Shinrin-yoku, a Japanese term that translates into English literally as forest bathing, refers to the exposure of individuals to forested environments [55] while “immersing oneself in nature by mindfully using all five senses” [25] (p. 851). In Japan, shinrin-yoku is a popular and recognised relaxation activity, especially since forests in Japan are easily accessible [28]. Such practice has been supported by a substantial amount of literature findings for its positive benefits, especially in the aspect of physiological health [25,33,55].

In a large-scale study conducted across 24 forests in Japan, Park, Tsunetsugu, Kasetani, Kagawa, and Miyazaki [56] examined the effects of shinrin-yoku through 24 experiments. Each experiment involved 6 male participants in each condition; taking a walk in the forest or the city area for approximately 15 min. On the second day, participants switched to the alternative condition. Results revealed a significantly lower salivary cortisol level, systolic blood pressure, diastolic blood pressure and pulse rate for participants after they took a walk in the forest as compared to taking a walk in the city area, and thus indicated greater physiological improvements derived from exposure to the forest environment than from the city environment.

Meanwhile, Song et al. [57] compared the efficacy of shinrin-yoku in sitting conditions. Twenty males sat on chairs and viewed either forest or urban landscapes. Results revealed that participants who viewed the forest landscape had significantly lower heart rates, indicating that they were calmer and more relaxed throughout the experimental condition.

In further support of the efficacy of shinrin-yoku, Hansen et al. [25] conducted a thorough review of research published in the period 2007–2017. Based on their closer examination of 64 studies referring to shinrin-yoku and/or forest bathing and/or nature therapy, they likewise concluded that shinrin-yoku reduces individuals’ heart rate and blood pressure, and increases relaxation levels. No differences were observed between participants of a different culture, gender, education, marital or economic status in the review.

Thus, these findings suggest that shinrin-yoku improves physiological functioning through forest exposure, as observed from the decrements in heart rate, blood pressure and salivary cortisol in related experimental studies.

Due to the wealth of research demonstrating the advantages of nature exposure, natural components have been adopted and developed as a part of treatment therapies, known as nature therapy. Nature therapy can be conducted in the form of animal-assisted therapy (AAT), horticulture therapy or forest therapy [28,58].

AAT refers to the use of animal interaction to produce therapeutic effects [59]. Horticulture therapy helps individuals gain an enhanced interest in their surroundings through hands-on activities such as gardening and weeding [60]. Forest therapy involves conducting therapeutic activities that expose individuals to a natural environment and can range from a garden walk to a wildlife expedition [56,58]. There is no fixed way of executing forest therapy, and its approach tends to vary across research studies.

For the current paper, we focus on studies that have examined the efficacy of forest therapy in the form of active engagement with the forest environment (meditation, walking, etc.).

Much of the research on forest therapy to date has emerged from Asian countries including Japan and South Korea. For example, Chun, Chang and Lee [61] recruited 59 stroke patients (40 males, 19 females); where half participated in a four-day-three-night forest therapy program at a recreational forest area in Gyenggi-do, Republic of Korea, and the other half formed the urban group who stayed at a hotel during the program duration. The program included activities such as meditation, experiencing the forest through the five senses, and walking in the forest. The urban group likewise engaged in the meditation and walking segments, but in an urban area. The majority (60–80%) of the stroke patients had depression and/or anxiety issues, and Chun et al. were interested to examine whether the forest therapy would be effective in improving mood and reducing stress among them. Results revealed that participants who went through forest therapy showed decreased scores on the Beck Depression Inventory (BDI), the Hamilton Depression Rating Scale and the Spielberger State-Trait Anxiety Inventory (STAI), indicating decreased levels in depression and anxiety. Meanwhile, participants from the control condition demonstrated increased scores in the STAI instead, indicating increased anxiety levels. This increase in anxiety could be due to feelings of unfamiliarity with the place of stay, and is attributable, at least partially, to living in an urban environment during treatment. Research has also shown that exposure to urban environments can bring about adverse effects to one’s mental health [62,63,64]. Thus, the findings reported by Chun et al. suggest that forest therapy is an effective tool to reduce depression and anxiety issues among stroke patients.

In another South Korean study, Shin, Shin and Yeoun [65] recruited and randomly assigned 92 alcoholics to partake in either a nine-day healing camp program in the forest or to the control condition where they proceeded with their usual daily routines. Depression levels were assessed using the BDI before and after the nine-day period. Activities during the camp included nature games, nature sports (mountain-climbing, tracking, etc.), nature meditation and counselling in the forest. Results revealed that participants who went through the healing camp experienced decreased levels of depression and had less difficulty falling and staying asleep, while depression scores of those in the control condition showed no changes.

Han et al. [66] recruited 60 participants with chronic widespread pain, who were assigned to either a forest therapy program condition or a control condition. In the forest therapy condition, participants completed an overnight camp at a forest in Yangpyeong, Korea. Activities included bodily exercises, mindfulness-based meditation, music therapy and more. For the control condition, participants were not engaged in any psychological or therapeutic treatments and were asked to proceed with their usual weekend routines while avoiding visits to natural environments and heavy occupational or domestic work. Results revealed that participants who went through the camp showed greater physiological relaxation, observed through an increase in heart rate variation, and greater activity in natural killer cells. A greater decrease in scores in depression and pain and a greater increase in health-related quality of life scores were found as compared to participants in the control condition.

Overall, findings seem to suggest that forest therapy brings about positive impacts on individuals’ psychological and physiological functioning, in line with the research literature on the benefits of nature exposure. However, it is important to note that for studies reported by both Shin et al. [65] and Han et al. [66], the control groups were not engaged in similar activities as the experimental groups. Thus, changes in physiological responses, affect and sleep could also be attributed to the novelty of the camp activities, as well as the relatively more fulfilling and exciting experiences for individuals who went through the nine-day healing camp program or the forest therapy program. Nonetheless, the results of the cited studies suggest that forest therapy can produce positive benefits among individuals with varying health issues.

Along with the increasing emergence of empirical support for potential benefits of shinrin-yoku and forest therapy, guided nature immersion programs have increased in availability and popularity. Recently in the USA, the Association of Nature and Forest Therapy Guides and Programs [67] has developed programs such as “The Council of Water and Trees” and “Seven Walks in Seven Weeks: Seasonal Change Immersions”, proposed to help individuals spend time in nature in a healing manner. This association alone reports having trained Forest Therapy guides “in 23 countries on 6 continents and there will be close to 450 certified guides by May 2019” [68].

The guided nature immersion programs follow closely with the ideals of shinrin-yoku and forest therapy and thus have great similarities with both. However, there are important and distinct differences to note as well. For shinrin-yoku, the practice emphasizes on immersing oneself in a forested environment, with no focus or suggestion of the engagement of activities during the immersion period. Forest therapy focuses on both the forested environment and activities involved, but there are no strict rules or guidelines for the duration, activity type and implementation style of the activities. As such, the procedures of forest therapy tend to vary largely depending on the user and therapist [52]. Meanwhile, guided nature immersions are led by trained forest therapy guides and there is usually a standard sequence that the programs follow, although slight variation may exist between programs.

A guided nature walk typically ranges between 2−4 h [67]. The guided walk usually begins with an “expression of gratitude” session, where participants share one thing that they are grateful for [52]. This helps to shift participants’ focus away from their daily worries. This is then followed by invitation 1 known as the “Pleasures of presence” that guides participants to focus on the present moment. Invitation 2 in the standard sequence is always “What’s in motion?” which invites participants to take notice and be aware of the motions of the natural environment. Invitation 3 (“It depends”) tends to vary, but generally focuses on sensory interactions with the natural environment, such as touching leaves or smelling flowers, and a guide might choose to include more than one of these “It depends” invitations in the sequence. The final invitation is usually called the “sit spot” phase, where participants reflect alone upon their overall experience. This latter phase can involve written reflection or merely a meditative reflection, and ‘sitting’ can be interpreted as standing still or slow pacing [52]. The walk ends on a ceremonial note with a tea ceremony. As observed, the standard sequence places a strong emphasis on the concept of mindfulness throughout the immersion experience, as participants are guided in various ways to focus on their sensory experiences in the present moment.

Mindfulness refers to the purposeful focus, awareness, and consciousness of the present moment in an open and non-judgmental manner [69]. Mindfulness is a tactic adopted in psychotherapy, often used in the form of meditation to help clients gain insights on themselves, the environment and the situation they are in, and to be less involved in rumination and instead focus on the present [70]. Such an approach has been found to be useful in the treatment of mental illness, assisting clients in their personal development and in seeking personal identity [58]. In the context of guided nature exposure programs, mindfulness emphasizes a focus on bodily sensations in the present moment and thus encourages individuals exposed to nature to be fully engaged in the process [71]. In her book, Nebbe [58] discusses nature therapy and reports that clients are often guided by trained psychologists to look within their inner minds and thoughts, to seek understanding and control. By inducing high internal state awareness and enhancing the richness and vitality of the moment-to-moment experiences, mindfulness allows the full nature experience to be attended to and appreciated with greater sensitivity, further strengthening the effect of nature on well-being [71].

Besides the concept of mindfulness, liminality is an alternative explanation for the possible enhancement of connectedness to nature and benefits of guided nature exposure. From a threshold concept and transformational learning perspective, Hansen, Jones and Tocchini [25] suggested that the practice of guided forest bathing leads individuals into a “liminal” space. Liminality was initially introduced as a term to describe the transition phase in rites of passage and commonly described as the suspended state of partial knowing [25,72]. This process is usually associated with feelings of fear and anxiety, as individuals must let go of their existing understanding of the world and enter a new phase that is filled with ambiguity and uncertainty [73].

According to Hansen et al. [25], in the context of nature exposure, individuals may experience a “pre-liminal” space with old and disturbing thoughts initially. However, once in the “liminal” psychological space, individuals would instead experience feelings of calmness and a sense of mastery. Hence, some researchers or experts propose that the effect of guided nature immersion may take place through engagement within the liminal state.

### 1.4. The Current Study

While there has been a rise in guided nature walk programs in the recent years and researchers positing its benefits and advantages, little has been known about the true effects of guided nature immersion; and particularly in areas beyond heavily forested environments such as in parks or botanic gardens. Specifically, there has been a lack of statistically controlled research experiments that compare the efficacy of a guided nature walk with that of the unguided nature walk. Most research findings examine the benefits of general nature exposure, others the benefits of shinrin-yoku or forest therapy, but few on guided nature immersion. As such, it is timely now to provide this needed evidence.

Findings could contribute to the literature on the possible benefits of guided nature immersion programs, and the advantages of these guided programs over unguided nature immersion experiences. If proven effective, the findings could potentially help to alleviate some of the demands on the healthcare system by helping community members develop stronger self-care strategies for psychological wellbeing.

As such, the current study aim was to compare the psychological benefits derived from a guided versus unguided nature immersion exposure. Specifically, the effects upon constructs of nature connectedness, mood and heart rate were examined.

**Hypothesis 1.**  *The two measures of nature connectedness (Connectedness to Nature Scale and Environmental Identity scale—short form) will be positively correlated.*

In the absence of recruitment bias, the initial level of nature connectedness can be expected to be similar between the two conditions. Thus, Hypothesis 2 is as follows:

**Hypothesis 2.**  *No between-groups difference in initial Connectedness to Nature and Environmental Identity scores will be observed.*

Literature has revealed that nature exposure improves mood, decreases anxiety and increases nature connectedness. With the practice of liminality and incorporation of mindfulness in the activities of the guided nature immersion experience, it can be expected that the benefits derived from nature exposure will be further strengthened. Thus, Hypotheses 3–5 are as follows:

**Hypothesis 3.**  *Participants of the guided nature walk will show greater improvement in nature connectedness than participants of the unguided nature exposure.*

**Hypothesis 4.**  *Participants of the guided nature walk will show greater improvement in mood than participants of the unguided nature exposure.*

**Hypothesis 5.**  *Participants of the guided nature walk will show a greater decrease in heart rate across the two-hour nature immersion period than participants of the unguided nature exposure.*

Among the five segments in the standard sequence, the first segment (Pleasures of Presence) involves activities that incorporate the most direct guidance towards a state of mindfulness. Thus, the effect of nature exposure is expected to be the strongest in this segment. With that, Hypothesis 6 is as follows:

**Hypothesis 6.**  *Participants going through the Pleasures of Presence (mindful sensory awareness) segment of the guided nature walk will experience the lowest average heart rate compared to other segments of the guided nature walk.*

## 2. Materials and Methods

### 2.1. Participants

Participants included students from the Singapore campus of an international university, as well as community residents of Singapore. Inclusion criteria for participation were as follows: Participants were either Singaporean nationals, Permanent Residents or those on a Singapore Employment Pass, above the age of 18 years, had normal or corrected to normal vision and were able to read and understand simple English. A total of 51 participants (age range: 20–66 years) were recruited, and Table 1 summarises the details of the sample.

### 2.2. Recruitment/Sampling Procedure

Ethics approval was gained from the university’s Ethics Committee before participant recruitment commenced (H7384). For students, information about the study was posted on the online SONA research management system [74]. In addition, a printed recruitment poster was displayed on the campus research noticeboard and a soft copy of the same poster was displayed on the university’s research web page. Meanwhile, for the recruitment of the community residents, flyers were distributed into letterboxes at residential areas proximal to Khoo Teck Puat Hospital (KTPH) (e.g., Yishun) where the study was conducted. Recruitment was also done through the Eventbrite webpage (one of the world’s largest event technology platforms) and a link to the Eventbrite listing was posted on the Facebook page of the certified forest therapy guide who conducted the guided walks.

In all forms of dissemination, information included a summary of the study, the required time commitment, the nature of the activity associated with the specific study, an offering of a small gift as an incentive, the nature of voluntary participation, assurance of anonymity of data responses and the investigators’ contact details. For incentives, eligible students received partial course credit for their participation, while other participants received a small memento with a value of approximately $2. On all platforms, interested individuals were directed to the Eventbrite webpage to register for a specific date for participation. A total of 16 sessions were organised on Thursdays and Fridays between July to September 2018.

### 2.3. Procedure

The Setting: It is important to the research aims that internal and ecological validity are retained. Forest therapy walks typically occur in outdoor, natural settings that are subject to external weather conditions and it is therefore difficult to control for consistency. To enable the evaluation of forest therapy across groups of people, it was important to control at least some of the external variables. The biophilic built design components of the Khoo Teck Puat Hospital (KTPH) public areas and the adjacent pathway around Yishun Pond Park offer a combination of natural settings that are external and yet semi-protected from the elements of weather, and shelter is close by if needed. Furthermore, the KTPH philosophy drawing on biophilic built design principles to assist in the healing process and to encourage community outreach aligns well with the philosophy of forest therapy, and the hospital management gave permission for the study to be conducted within the publicly accessible garden areas on the hospital grounds.

Allocation: All sessions were conducted on either Thursday or Friday, 8 am to 11 am. The guided nature immersion sessions were conducted on Thursdays, while the unguided sessions were conducted on Fridays. Participants were only aware of the nature of their allocated session upon arrival for the session. The final two Friday sessions were changed into guided sessions to increase the number of participants for the guided condition.

Upon sign-up, participants would receive a confirmation email detailing information such as specific meet-up location and things to bring. A reminder email with the same content was also sent to participants one day before each session. A maximum of eight participants was allowed for each session due to the number of wristwatch trackers available for use.

Preliminaries: Participants were gathered together at the study site for a preliminary briefing. After a simple introduction, each participant was given an information sheet detailing the study background, aim, and participation requirements. The information sheet varied depending on whether participants were in the guided or unguided condition. The information sheet for the unguided condition had an additional page indicating a list of suggested activities for the participants and a map of the KTPH area.

Once all participants had arrived for a specific session, the chief investigator (CI) provided a brief summarisation of the study. An informed consent form was given to each participant to seek their consent to participate in the research study and to allow photographs of themselves to be taken and uploaded to social media sites (the latter a condition of the funding body). Signed consent forms were collected and stored in a sealable container for safekeeping until securely stored at the university campus. Consent forms were kept separately from study data and paperwork required by the funding body.

Upon participants providing signed consent, the questionnaire booklet was distributed. The booklet contained questions from the Connectedness to Nature Scale (CNS), Environmental Identity scale short form (EID), Positive and Negative Affect Scale (PANAS), Good Health Practice scale (GHP), and some demographic questions, as well as open-ended questions about the participant’s overall experience of the nature immersion. The pre-test included items from the CNS, EID and PANAS. The post-test was identical to the pre-test, but with the addition of the GHP, demographic questions and open-ended questions. Both pre-test and post-test items were collated into the one booklet, with a blank page separating the two sections and a note to participants not to proceed further until the nature immersion experience was completed.

Each questionnaire booklet was coded with an identifier linked to the participant to ensure anonymity. After completion of pre-test items, the CI collected questionnaire booklets and fitted each participant with a heart rate tracker watch (coded with the same identifier). Upon fitting of the heart rate tracker, participants were asked to hand in their National Republic Identity Card (NRIC) as a security deposit to be reclaimed upon return of the tracker watch. The CI was responsible for handling all preliminary activities relevant to the research study, including the fitting of wristbands, on-site security and the return of NRICs and distribution/collation of forms and questionnaire measures. Participants were asked to avoid fiddling with or touching the screen of the watch, in case of accidentally stopping the tracking function. A break time of 5 min was allocated for participants to visit the washroom if required. Insect repellent was also passed around for use. As participants in the guided condition would be using a yoga mat for sitting purposes during two of the guided sequence components, participants from the unguided condition were offered yoga mats to take along with them for their nature immersion if they desired.

Participation: Once all participants were fitted with a wristband and had completed pre-test questionnaires, the forest therapy guide briefed participants to begin the sequence for the guided format.

For the unguided format, the guide delivered the same set of introductions before leaving participants to follow a printed list of suggested activities during their nature exposure session (suggested timing approximately 2 h duration). Suggestions included exploring the gardens slowly while keeping an eye out for sensations such as movements of fish or birds, textures of trees/shrubs, feeling the wind on skin, listening for sounds far away and close up, and smelling the air in different places. A final suggestion was to sit quietly while taking in surroundings at various spots. These suggested activities were similar to that of the guided format but differ in the absence of a trained guide to guide participants through the immersion process. Participants in the unguided sessions were directed to return the heart rate tracker to the CI after their 2 h nature immersion experience (e.g., upon completion of their chosen activities). The CI remained available at a designated place at the study site until all trackers were returned and NRICs reclaimed. Upon return of the tracker, each participant was asked to complete a second set of questionnaire items coded with their unique identifier.

For the guided format, the certified forest therapy guide led participants through the standard sequence (approximately 2 h) of a Guided Forest Therapy Walk. For the purposes of the current study, the sequence consistently followed Pleasures of Presence, What’s in Motion, It Depends 1 (Water gazing), It Depends 2 (Tree befriending), and Sit Spot followed by the Tea Ceremony as a formal ending to the walk sequence. The CI and RA joined during the social component of the tea ceremony (after the formal ending of the walk sequence) to collect the trackers/return of NRIC and invite participants to complete of the second set of questionnaire items. Upon completion of the second set of questionnaires, the CI was responsible for collating all the response sheets.

Debriefing: Participants were thanked for their participation in the research study, offered the opportunity to ask questions about the study and were informed about the opportunity to attend a university conference to learn about study outcomes. Public participants were given a small gift to thank them for their participation, while students either received partial course credit for their participation or a small gift.

### 2.4. Measures

The pre-test and post-test were conducted in pen-and-paper format. Demographic questions were collected together with post-immersion measures. There were three demographic questions, namely age and gender, and a question enquiring on the participants’ frequency of ‘nature experience that is longer than an hour’, for the past one year. To obtain a general idea of participants’ experience of the nature walk, one post-immersion open-ended question was asked “Compared to how you felt before you began the nature immersion experience today, do you notice any changes in how you feel now?” There were also three post-immersion open-ended questions with the first question expanding upon the closed-ended question above—(1) If yes, describe the changes, (2) In your own words, how would you describe your typical nature immersion experiences? (e.g., In wilderness areas? In National Parks around Singapore? In safe/challenging environments?) and (3) In your own words, how would you describe your nature immersion experience today?

To assess the individuals’ emotional connection with nature, the Connectedness to Nature Scale (CNS) [75] was adopted. The scale consists of 14 items, and all items are based on a 5-point Likert scale ranging from “1—strongly disagree” to “5—strongly agree”. According to Mayer and Frantz [75], the CNS has shown good internal consistency, with a Cronbach’s alpha of 0.84. The CNS has also been found to be highly correlated with alternative scales such as the New Ecological Paradigm (NEP) scale [75].

To assess individuals’ environmental identity, the Environmental Identity scale (EID)—short form was adopted (Personal email communication, Clayton, June 2016). The shorter form consists of 11 items as compared to the original scale of 24 items [36]. The scale rating is based on a 7-point Likert scale ranging from “1—Not at all true of me” to “7—completely true of me”. The EID—short form showed good internal consistency with a Cronbach’s alpha of 0.88 in a European sample [76]. An EFA also supported the unidimensional structure of the EID short form and a Cronbach’s alpha of 0.92 in a Singaporean sample [77]. The scale also showed a medium-strong correlation of 0.57 with a similar scale assessing nature connectedness — the Inclusion of Nature in Self (INS) scale developed by Schultz [35].

To assess the participants’ mood, the Positive and Negative Affect Scale (PANAS) was used [78]. The questionnaire consists of 20 items, with 10 items assessing positive affect (PA) and 10 items assessing negative affect (NA). All items are based on a 5-point Likert scale ranging from “1—very slightly or not at all” to “5—extremely”. The Cronbach’s alpha for PA ranges from 0.86 to 0.90, and 0.84 to 0.87 for NA, thus indicating good internal consistencies and construct validity [78,79]. The NA scale also correlates strongly with alternative measures such as the Hopkins Symptom Checklist (HSCL) that measures anxiety and depression [78]. On the other hand, the PA scale showed a modest negative correlation with the HSCL. The NA scale is substantially correlated with the Beck Depression Inventory (BDI) that assesses depression scores, and the PA scale has a significant negative correlation with the BDI [78].

To assess participants’ general health condition, the Good Health Practice scale (GHP) was used. The GHP scale is a shortened, revised version of the Health Behaviour Checklist [80]. The scale was used to capture cardiovascular and metabolic risk factors associated with heart rate, which is an important measure examined in this study. Questions of the scale include statements such as “I don’t smoke” and “I see a doctor for regular check-ups”. Participants respond by using a 5-point Likert scale ranging from “1—Not at all like me” to “5—Very much like me”. The GHP scale items are categorised into 3 subtypes, good congruency was observed within each subtype of items; Good Health Practices (0.92), Risk Avoidance (0.95), and Other Health Concerns (0.85) [80]. All except one item are significantly correlated with the assessment of physiological dysregulation (based on a composite of 11 biomarker measures.

Heart rate: To assess participants’ heart rate as a measure for physiological changes, nine TomTom Touch Cardio Fitness Tracker watches were purchased. These wristwatch trackers have been found to be highly accurate in measuring heart rate [81]. In a study conducted by Stahl, An, Dinkel, Noble and Lee [82], the TomTom Runner Cardio was found to have the lowest mean absolute percentage error of 3.3% among five other heart rate wristwatch trackers. As the GPS function of the TomTom Runner Cardio was not required for the purposes of the current study, the TomTom Touch Cardio that works similarly but cost less was chosen for use. The tracker assesses the heart rate by shining a light through the skin and onto one’s capillaries to measure changes in the blood flow. The tracker assesses the heart rate at every second upon activation of “sports mode”. The exact time (to the second) that the “sports mode” was activated and deactivated was recorded by the CI. In addition, the start and end time of each component of the guided sequence was recorded to allow for comparison of mean heart rate level between components.

### 2.5. Design

This is a mixed-method feasibility study using quantitative and qualitative research methodologies. The Connectedness to Nature Scale (CNS), Environmental Identity (EID) scale, Positive and Negative Affect Scale (PANAS) and heart rate were administered pre- and post-intervention delivery (T1, T2). Frequency of nature immersion experience, demographics and general health practice (GHP) were assessed once post-intervention delivery. Three qualitative questions were used to obtain written participant reflections after the intervention session.

All statistical tests were run using IBM SPSS 20 (IBM Corp. in Armonk, NY, USA). Nature connectedness and mood data were analysed with a 2 (Time: Pre versus Post) × 2 (Conditions: Guided versus Unguided) mixed ANOVA (positive and negative mood analysed, separately). For heart rate data, a one-way randomised ANCOVA was used to compare the average heart rate between the two conditions (using pre-activity resting heart rate as a covariate), while a repeated-measures ANOVA was used to compare the average heart rate between segments of the guided condition. Qualitative data were analysed through thematic analysis to explore themes that arose from participants’ reflections.

## 3. Results

### 3.1. Data Entry

Questionnaire booklet: All responses from the questionnaire booklet were manually keyed into Microsoft Excel 2016 by the CI. One item of the CNS for both pre-test and post-test was reverse coded.

Heart rate data: All heart rate data were downloaded from the respective wristwatch account from the TomTom website. The relevant heart rate data (i.e., within the period of specifically recorded outset-cessation times unique to each session) were extracted and transferred into an Excel sheet along with the respective participant code to be matched with questionnaire data. The average heart rate was calculated by averaging the heart rate data from the start to stop-time (to the exact second) for each participant’s nature immersion experience. The baseline heart rate was calculated by averaging the 10 min of heart rate data immediately before the start-time (a period where participants were given toilet break-time and introduction brief by nature guide). For the guided condition, the start and end time of each segment was recorded as the CI was present as an unobtrusive observer during the nature immersion, for every guided session. This timing was used to segment the heart rate data for each participant from the guided condition, and the average heart rate for each segment was calculated.

Once the data compilation of the questionnaire responses and heart rate data was complete, the data set was transferred to IBM SPSS 20 for analyses. All subsequent data reporting is based on a 95% confidence interval.

### 3.2. Quantitative Data

Data Screening: All measures were reviewed for completeness or missing data. One participant did not complete the entire GHP scale and both the pre-test and post-test for PANAS but did, however, complete other questionnaires, which were included for analyses. In addition, two participants accidentally stopped the heart rate tracking during the early phases of the nature immersion, and their entire heart rate data were thus removed from analyses. Data were screened for violation of assumptions prior to the analyses.

All measures were screened for outliers, and only the PANAS negative affect response set contained outliers. For the pre-test, there was one outlier and two extreme outliers, while in the post-test, there was one outlier and one extreme outlier. The extreme outliers for pre-test scored 3.2 and 2.7, and the extreme outlier for post-test was 2, out of a 5-point Likert scale; representing moderate negative affect. However, the reason these scores were labelled as extreme outliers was due to the negatively skewed response set for both pre-test (M = 1.26, SD = 0.41) and post-test (M = 1.14, SD = 0.21), indicating that most participants experienced extremely low negative affect before and after the nature immersion experience. This extreme skewness made an average score appear as an extreme outlier. Thus, the outliers were not removed as the data do not suggest experimental error but, rather, normal variation in the measure.

Cronbach’s alpha of the pre-test for each measure was also calculated, with all results indicating good internal consistency; CNS (α = 0.87), EID (α = 0.88), PANAS (positive mood, α = 0.75; negative affect, α = 0.84) and GHP (α = 0.85). One participant in each session wore two wristwatch trackers to assess the reliability of the heart rate tracking. Comparison between the repeated trackers revealed high reliability (α = 0.97).

Extraneous Variables: It was important to ensure that there were no between-group differences on extraneous factors of nature exposure frequency and general health practices that could potentially influence the results. Thus, between-group comparisons were done upon these two extraneous variables.

Frequency of nature immersions: A one-way between-groups analysis of variance (ANOVA) was used to examine if there were any significant differences between the frequency of nature immersion experiences for the past one year between participants in the guided or unguided conditions.

Inspection of the skewness and kurtosis indicated that the assumption of normality was supported for both guided and unguided conditions. The Shapiro–Wilk statistic was non-significant for the unguided condition but was statistically significant for the guided condition, *p* = 0.02. However, research has suggested that ANOVA is robust against violations of normality, and thus the analysis proceeded despite the violation [83,84]. Levene’s statistic was non-significant, F (1, 49) = 2.26, *p* = 0.139, and thus the assumption of homogeneity of variance was not violated.

The ANOVA was statistically non-significant, indicating that there are no differences between the frequency of nature immersion experiences for the past one year between participants from the guided or unguided condition, F (1, 49) = 0.02, *p* = 0.887.

GHP: A one-way between-groups ANOVA was used to examine if there were any significant differences between the general health practices (GHP) scores between participants from the guided or unguided condition.

Inspection of the skewness, kurtosis and Shapiro–Wilk statistics indicated that the assumption of normality was supported for both guided and unguided conditions. Levene’s statistic was non-significant, F (1, 48) = 0.14, *p* = 0.711, and thus the assumption of homogeneity of variance was not violated.

The ANOVA was statistically significant, indicating that there was a difference between the general health practices between participants from the guided versus unguided condition, F(1, 48) = 12.97, *p* = 0.001. Participants from the unguided condition had a significantly lower average GHP score (M = 2.69, SD = 0.62) than participants from the guided condition (M = 3.37, SD = 0.69).

As GHP differed significantly between the two conditions, further tests were conducted to examine the relationship between GHP and the dependent variables. Scatterplots of GHP with each of the dependent variables demonstrated a non-linear relationship. This is further supported by findings from a 2 (Time: Pre versus Post) × 2 (Conditions: Guided versus Unguided) mixed ANCOVA model analysis that was conducted for each of the dependent variables of CNS, EID, PANAS (positive and negative mood analysed separately) and heart rate, with GHP as the covariate for all five analyses. Results revealed there was no significant correlation between the covariate (GHP) and all five dependent variables. Results of the analyses are summarised in Table 2 below. In other words, despite the difference in GHP scores between the conditions, ANCOVA results suggested that this difference was not a cause for concern as GHP does not correlate with the dependent variables assessed in the current study. Thus, the data analyses went as per initially planned, without a need to consider the effect of participants’ general health practices.

Hypothesis 1: To assess the size and direction of the linear relationship between pre-test CNS and EID scores, a bivariate Pearson’s product-moment correlation coefficient (*r*) was calculated. The bivariate correlation between these two variables is significantly positive and moderate, *r*(49) = 0.61, *p* < 0.01.

Before the analysis, the assumptions of normality, linearity and homoscedasticity were assessed and found to be supported. Specifically, a visual inspection of the normal Q-Q and detrended Q-Q plots for each variable confirmed that both were normally distributed. Similarly, visually inspecting a scatterplot of CNS scores against EID scores confirmed that the relationship between the two variables was linear and heteroscedastic.

Hypothesis 2: A one-way between-groups ANOVA was used to investigate if there was any statistical difference in CNS scores between the guided and unguided conditions. Besides the kurtosis of scores for the unguided condition, inspection of skewness, and Shapiro–Wilk statistics indicated that the assumption of normality was supported for both conditions. Since the Shapiro–Wilk statistic is non-significant and indicates no violations of normality, the ANOVA proceeded as normal. Levene’s statistic was non-significant, *F*(1, 49) = 3.65, *p* = 0.062.

The ANOVA was statistically non-significant, *F*(1, 49) = 0.09, *p* = 0.765, confirming no significant difference in initial CNS scores between the guided and unguided conditions.

A one-way between-groups ANOVA was used to investigate if there was any statistical difference in EID scores between the guided and unguided conditions. Inspection of the kurtosis, skewness, and Shapiro–Wilk statistics indicated that the assumption of normality was supported for both conditions. Levene’s statistic was non-significant, *F*(1, 49) = 0.23, *p* = 0.635.

The ANOVA was statistically non-significant, *F*(1, 49) = 0.003, *p* = 0.957, confirming no significant difference in initial EID scores between the guided and unguided conditions.

Hypothesis 3: A 2 (Time: Pre versus Post) × 2 (Conditions: Guided versus Unguided) mixed ANOVA model was used to compare between participants’ Connectedness to Nature Scale (CNS) score before and after the nature immersion experience, between the two conditions.

The Shapiro–Wilk, F_max_ and Levene’s test statistics were used to test the assumptions of normality and homogeneity of variance. The assumptions for a mixed model ANOVA were not violated.

A significant main effect for time was obtained, *F*(1, 49) = 37.41, *p* < 0.01, partial *η*^2^ = 0.43, with the post-test CNS score (*M* = 3.71, *SD* = 0.48) being significantly higher than the pre-test (*M* = 3.45, *SD* = 0.52). There was no significant interaction effect between CNS change and condition, *F*(1, 49) = 0.02, *p* = 0.896, partial *η*^2^ < 0.001.

A 2 (Time: Pre versus Post) × 2 (Conditions: Guided versus Unguided) mixed ANOVA model was used to compare between participants’ Environmental Identity (EID) score before and after the nature immersion experience, between the two conditions (guided versus unguided).

The Shapiro–Wilk, *F*_max_ and Levene’s test statistics were used to test the assumptions of normality and homogeneity of variance. The assumptions for a mixed model ANOVA were not violated.

**Figure 1:**
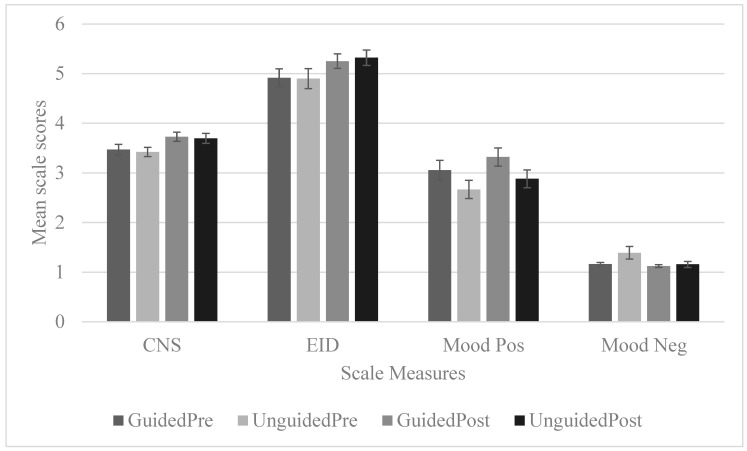
Mean scores on pre-test and post-test on four measures for Guided versus Unguided conditions. Error bars represent standard errors.

A significant main effect for time was obtained, *F*(1, 49) = 18.78, *p* < 0.01, partial η^2^ = 0.28, with the post-test EID scores (*M* = 5.28, *SD* = 0.95) being significantly higher than the pre-test scores (*M* = 4.9, *SD* = 0.52). There was no significant interaction effect between EID change and condition, *F*(1, 49) = 0.23, *p* = 0.634, partial *η*^2^ = 0.005. Figure 1 displays comparative mean scores for guided and unguided conditions for pre- and post-tests.

Hypothesis 4: A 2 (Time: Pre versus Post) × 2 (Conditions: Guided versus Unguided) mixed ANOVA model was used to compare between participants’ positive affect before and after the nature immersion experience, between the two conditions (guided versus unguided).

The Shapiro–Wilk, *F*_max_ and Levene’s test statistics were used to test the assumptions of normality and homogeneity of variance. The assumptions for a mixed model ANOVA were not violated, except for the Shapiro–Wilk statistics for the pre-test, *p* = 0.021. However, as proposed by the work of Glass et al. [83] and Schmider et al. [84], ANOVA testing is rather robust against violations of normality. Hence, the mixed model ANOVA analysis proceeded as planned.

A significant main effect for time was obtained, *F*(1, 48) = 4.78, *p* = 0.034, partial *η*^2^ = 0.09, with the post-test positive affect scores (*M* = 3.14, *SD* = 0.94) being significantly higher than the pre-test scores (*M* = 2.9, *SD* = 0.99). There was no significant interaction effect between positive affect change and condition, *F*(1, 48) = 0.05, *p* = 0.816, partial *η*^2^ = 0.001.

A 2 (Time: Pre versus Post) × 2 (Conditions: Guided versus Unguided) mixed ANOVA model was used to compare between participants’ negative affect before and after the nature immersion experience, between the two conditions (guided versus unguided).

The Shapiro–Wilk, *F*_max_ and Levene’s test statistics were used to test the assumptions of normality and homogeneity of variance. The assumptions for a mixed model ANOVA were violated, *F*_max_ is 13.76, Levene’s test for both pre-test (*p* = 0.009) and post-test (*p* = 0.035) were significant, and the Shapiro–Wilk statistics for both pre-test and post-test were significant as well, *p* < 0.01 for both. Transformation of data (*Z* score normalization to produce a more normal distribution, squaring the data to reduce negative skewness and removal of outliers) was attempted to address the issue of homogeneity and normality violation. However, more outliers emerged while assumptions remained violated. As such, the original data were used, and a more conservative *p*-value of 0.01 was adopted for this analysis instead to counter the issue. Outliers were not removed from the original set of data for reasons detailed in the section labelled ‘data screening’ above.

The ANOVA analysis revealed a significant main effect for time, *F*(1, 48) = 8.48, *p* = 0.005, partial *η*^2^ = 0.150, with the pre-test negative mood scores (*M* = 1.26, *SD* = 0.41) being significantly higher than the post-test scores (*M* = 1.14, *SD* = 0.21). There was no significant interaction effect between negative affect change and condition, *F*(1, 48) = 4.4, *p* = 0.041, partial *η*^2^ = 0.084.

Hypothesis 5: A one-way between-groups analysis of covariance (ANCOVA) was initially proposed to compare participants’ average heart rate between the two conditions (guided versus unguided), using pre-activity resting heart rate as a covariate. However, a scatterplot of the covariate (pre-activity heart rate) and the dependent variable (average heart rate throughout nature immersion experience) revealed that there was no linear relationship between the two variables. This violates the assumption of an ANCOVA analysis where the relationship of the covariate and dependent variables have to be linear for the test to proceed. Further, a one-way ANOVA that compares participants’ baseline heart rate between the two conditions revealed no significant difference, *F*(1, 47) = 0.58, *p* = 0.450.

Hence, a one-way between-groups ANOVA was used instead to compare participants’ average heart rate between the two conditions (guided versus unguided).

Inspection of skewness, kurtosis and Shapiro–Wilk statistics indicated that the assumption of normality was supported. Levene’s statistic was non-significant, *F*(1, 47) = 0.18, *p* = 0.671

The ANOVA was statistically non-significant, indicating that participants’ average heart rate did not differ significantly between the guided and unguided conditions, *F*(1, 47) = 0.08, *p* = 0.782.

Hypothesis 6: A one-way repeated measures ANOVA was used to compare guided participants’ baseline heart rate and average heart rate across the five segments of the guided nature immersion.

The results of kurtosis for all groups of data were normal. The results of skewness and Shapiro-Wilk were normal except for segment 1 and segment 2, suggesting that normality was violated for the two groups, and data were skewed. Once again, research has demonstrated that the ANOVA test is robust against the normality assumption, and the analysis thus proceeded as planned. *F*_max_ is 1.37, indicating that the homogeneity of variance has not been violated. The Mauchly’s test of sphericity has been violated, *p* < 0.01. Hence, the Epilson adjusted test of Greenhouse–Geisser statistics are used for reporting instead.

**Figure 2:**
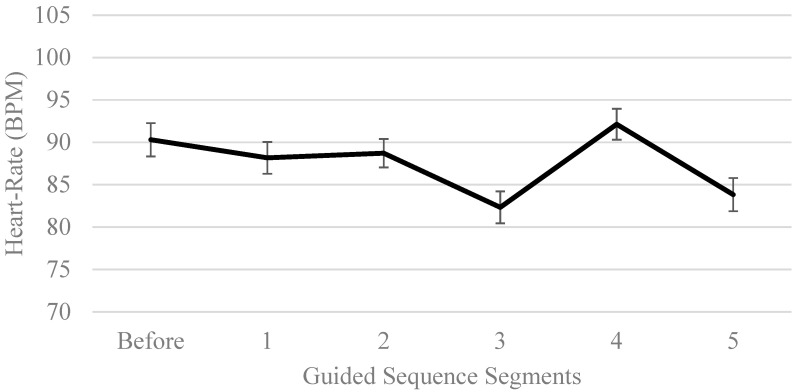
Averaged heart-rate (*n* = 37) for Baseline (Before) and each of the five guided sequence segments: (1) Pleasures of Presence, (2) What’s in Motion, (3) It Depends 1 (Water gazing), (4) It Depends 2 (Tree befriending), and (5) Sit Spot. Error bars represent standard errors.

The ANOVA results showed that heart rate was statistically different in at least 2 segments, *F*(5, 140) = 16.48, *p* < 0.01, partial *η*^2^ = 0.371. Planned simple contrast comparisons were performed to examine the change in heart rate from the baseline heart rate. Results revealed that the heart rate among guided participants in segments 3 and 5 were statistically different from the baseline heart rate, but not for segments 1, 2 and 4. The broad overview of heart rate change across segments can be observed in Figure 2.

### 3.3. Qualitative Data

Three open-ended questions were asked in the post-test to obtain a general idea of participants’ experience of the nature immersion.

For the first question, several participants appeared to have misunderstood the question and described their nature immersion experience during the research session, instead of their past nature immersion experience. In addition, it was difficult to entirely filter out the irrelevant responses as the majority of the responses were ambiguous as to whether they described the past or current nature exposure experience. As such, the responses for question one are not reported here.

**Figure 3:**
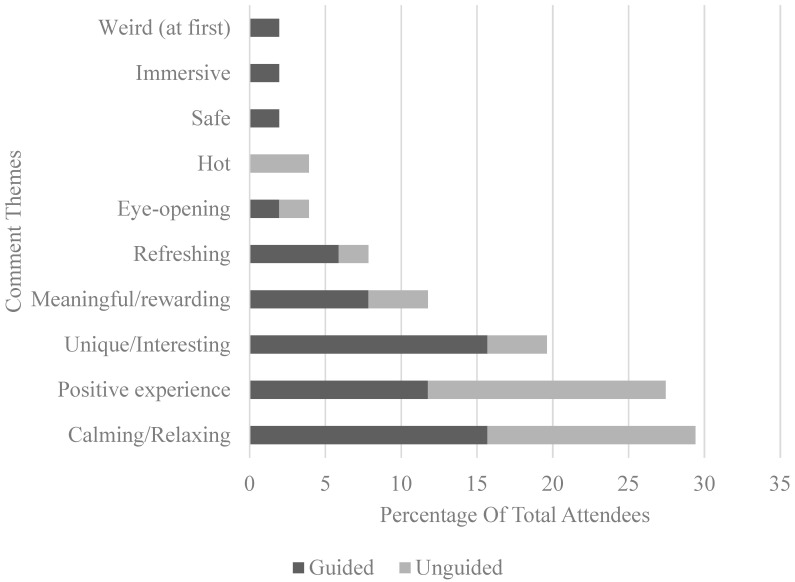
Descriptions of the nature immersion experience. Not all participants responded to open-ended items, so percentages do not represent the entire sample.

**Figure 4:**
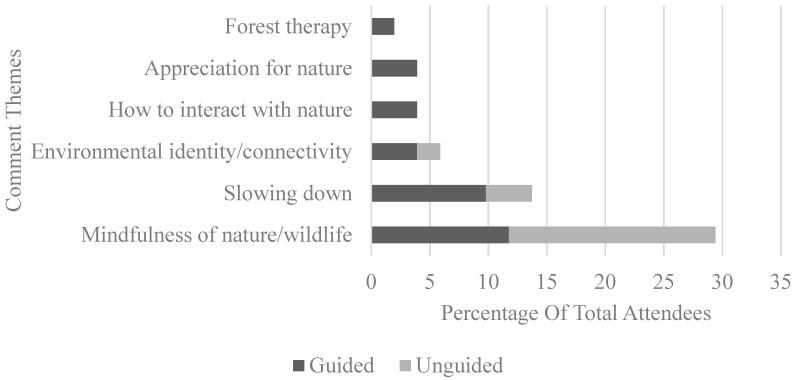
Reported learnings and/or improvements gained from the nature immersion session. Not all participants responded to open-ended items, so percentages do not represent the entire sample.

For the second question, two main categories of responses were observed among the 51 participants (30 guided, 21 unguided), namely the description of the immersion experience and things learnt from the experience. Common themes were drawn out and are summarised in Figure 3 and Figure 4 respectively.

**Figure 5:**
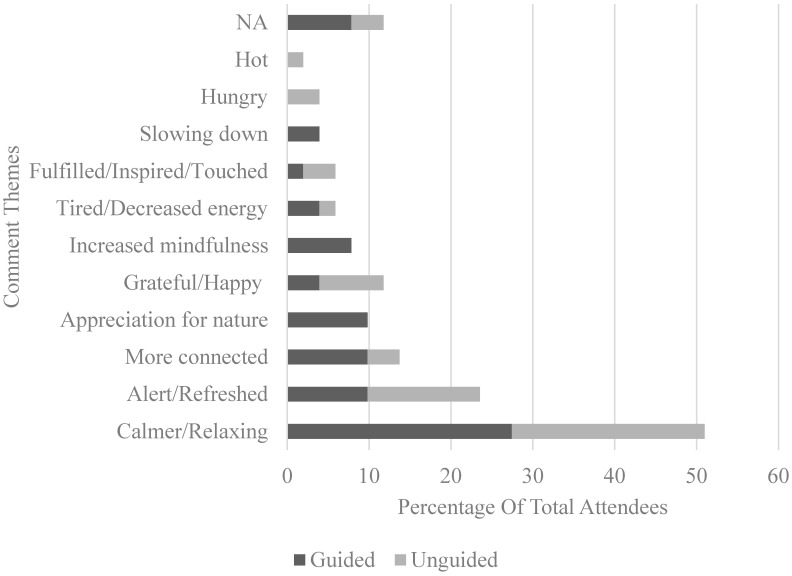
Changes that participants reported feeling after the nature immersion experience. Not all participants responded to open-ended items, so percentages do not represent the entire sample.

For the third question, common themes of the changes that the 51 participants (30 guided, 21 unguided) observed in themselves were drawn out and summarised in Figure 5.

## 4. Discussion

Even though numerous studies have tested the efficacy of forest therapy experiences that included similar components/activities as in the standard sequence (e.g., mindfulness meditation in the forest), there has yet to be a published study that has applied an intervention of forest therapy with the full standard sequence [52,61,65,67]. Current literature suggests that forest therapy is an effective intervention to reduce anxiety and improve mood. Studies on nature connectedness have also suggested that exposure to nature can improve individuals’ connectedness to nature. In addition, mindfulness that is highly incorporated in guided nature immersion programmes is expected to further strengthen the effect of nature exposure on well-being. Thus, the current study aim was to compare between the efficacy of guided versus unguided nature immersion experience, and hypotheses were proposed to test whether participants from the guided condition would experience a greater increase in nature connectedness and mood, and a lower average heart rate after the immersion experience, compared to participants from the unguided condition. Comparing between the segments of the guided condition, it was expected that the participants would experience the lowest average heart rate in the first segment compared to other segments. No significant difference in pre-immersion connectedness to nature and environmental identity was expected between conditions, and the two measures were hypothesized to correlate positively.

### 4.1. Quantitative Data

Hypothesis 1: Between-group ANOVA analyses of CNS and EID scores revealed that participants did not differ significantly in their initial nature connectedness score before interventions were implemented, supporting the first hypothesis. This shows that nature connectedness was homogeneous between participants from the two conditions prior to nature immersion.

Hypothesis 2: The two measures of CNS and EID were found to be positively correlated, thus indicating convergent validity in their measure of nature connectedness, supporting the second hypothesis.

Hypothesis 3: The mixed model ANOVA analysis revealed that participants’ levels of nature connectedness increased after the nature immersion experience, but this increment did not differ significantly between the guided and unguided conditions. In other words, the increment in nature connectedness was similar for the unguided and guided conditions. This finding did not support the third hypothesis, where participants of the guided nature walk were expected to show greater improvement in nature connectedness as compared to participants of the unguided nature exposure.

Hypothesis 4: The mixed model ANOVA analysis revealed that the positive effect scores increased while negative affect decreased after the nature immersion experience. This shows that mood improves after the nature immersion experience. However, the changes in mood did not differ between the guided and unguided conditions. Thus, the finding did not support the fourth hypothesis, where participants of the guided nature walk were expected to show greater improvement in mood as compared to participants of the unguided nature exposure.

Hypothesis 5: The ANOVA analysis revealed that the average baseline heart rate did not differ significantly from the average heart rate across the entire nature immersion experience. In other words, there was no significant change in heart rate due to nature immersion. Neither was there any significant difference in the average heart rate between participants in the guided and unguided conditions. This finding did not support H5, where participants of the guided nature walk were expected to show a lower average heart rate as compared to participants of the unguided nature exposure.

Explaining findings from Hypotheses 3–5. Taken together, findings from Hypotheses 3–5 are in line with the literature, showing that nature exposure leads to increment in levels of nature connectedness and mood. However, when compared between the conditions, there was no difference in nature connectedness, mood and heart rate between the guided and unguided conditions. This finding contradicts the propositions of researchers and organisers of guided nature walk programs, who suggested that the impact of nature upon the three variables will be further strengthened through the incorporation of mindfulness activities in nature.

One plausible explanation could be that the difference in the effect of nature exposure between the guided and unguided conditions is relatively small due to several following reasons, making it difficult for the current study to identify this difference.

First and foremost, it is important to note that the sample size (51 participants) was less than optimal for the planned analyses. An a priori analysis using G*Power [85] indicated a sample size requirement of 98 participants for a 2 × 2 mixed model ANOVA and 102 participants for a one-way between-groups ANOVA. Despite extensive recruitment attempts, the sign-up rate for the research sessions remained low. Furthermore, due to restrictions of the schedule and availability of the forest therapy guide and the restrained time period for the honours thesis project, there was no opportunity to implement more research sessions or extend the period of data collection for the current study. Thus, outcomes must be considered in light of the less-than-optimal sample size.

Secondly, it is important to note that research studies that have examined and identified effective changes from forest therapy tended to use programs that lasted for days. Specifically, Chun et al. [61], Shin et al. [65] and Han et al. [66] employed forest therapy programs lasting for four days, nine days and two days respectively. In contrast, in the current study, the nature immersion experience lasted for approximately two hours for both conditions, which could have led to the effect of nature exposure being smaller than what was observed in other studies. Likewise, with the availability constraints of the forest therapy guide, and other time constraints for the current study, there was no opportunity to implement a study design that involved conducting numerous sessions for each participant. Furthermore, as the current study focuses on a guided nature immersion program that adhered to the standard sequence for consistency, it would not be feasible to extend the duration of the immersion program to any great extent.

Thirdly, as mentioned in the introduction section, research has revealed that nature exposure leads to greater nature connectedness, and level of nature connectedness has been found to mediate the effects of nature exposure [39,43,44]. In the current sample, it seems that participants did not have a very high frequency of prior nature exposure, nor a high nature connectedness score. For the item that asked about participants’ frequency of nature exposure for the past one year, participants gave an average rating of 3, translating to 5–6 nature immersion experiences for the past one year. This reflects a low frequency of nature exposure. Comparing participants’ average CNS score in the current study with scores from another study, it seems to be much lower as well. A study by Mayer et al. [44] in the USA compared to participants’ CNS scores after being exposed to nature versus an urban environment. Mayer et al. reported the average post-test CNS scores for participants in nature and urban exposure conditions were 4.69 and 3.73, respectively, while the average post-test score of CNS for the guided and unguided conditions in the current study were 3.73 and 3.70, respectively. This indicates that current study participants experienced generally low levels of connectedness to nature in general, possibly due to the low frequency of nature exposure amongst the sample. With nature connectedness as a mediator of the effects of nature exposure, it is possible that the effect of the immersion experience would be smaller than what was typically observed in other studies.

Due to factors such as low nature connectedness in the sample and shorter duration of nature immersion experience compared to other studies, it can be expected that the effect of nature exposure might be smaller and more difficult to detect. As a result, an actual, existing effect could have gone undetected due to these factors.

Alternatively, it could also be possible that there are no additional benefits from having a guide in a nature immersion experience as compared to an unguided experience.

Hypothesis 6: For Hypothesis 6, comparisons of the segments of the standard sequence in the guided condition revealed the lowest average heart rate occurred during segments 3 (Water gazing) and 5 (Sit spot). Further, only these two components were significantly different from the baseline heart rate. While this contradicts the fifth hypothesis where the lowest heart rate was expected to be from segment 1 (Pleasures of presence), it is important to note that participants were seated down when engaged in activities of segments 3 and 5, and were standing/walking around during segments 1, 2 and 4. This difference in activity type is likely to explain the contradictory finding. Furthermore, comparing between standing/walking segments of 1, 2 and 4, the average heart rate of segment 1 was lower than that of segment 2 and 4, although they are not statistically different. Once again, this non-significant difference could be due to the small sample size, short duration of nature exposure and low CNS score. Further exploration employing repeated exposure, a larger sample and a sample with greater nature connectedness are needed to validate current findings.

### 4.2. Qualitative Data

Across the themes that emerged from the qualitative data, the majority of the participants reported feeling more relaxed, refreshed, and happy after the nature immersion experience, whether guided or unguided. This can be observed from comments such as “I felt more relaxed after the experience. I also felt more alert” and “feel more relaxed, happy and proud of this experience. I want to do it again anytime I can. I am strongly convinced that nature inspires me and makes me feel so comfortable”. Participants from both conditions reported an increase in mindfulness, connectivity with nature, and learning to slow down during their nature walk. Examples of such responses include “A lot of mindful observations. Prompts allow us to focus our attention to specific elements of the surroundings” and “Relaxing, as usually we won’t find time to enjoy this ‘slower pace’ of life and to observe our surrounding of nature”. However, these gains did not seem to differ between the two conditions as well, in line with the quantitative findings.

### 4.3. Theoretical and Practical Implications

While findings are not in line with most of the hypotheses, the findings of the current study are nonetheless a good start to examining the efficacy of guided nature immersion programs and can guide future related research with respect to the methodological design.

In addition, the benefits of nature exposure are once again reinforced, and findings suggest that even built venues incorporating biophilic built design features, such as KTPH, can be suitable locations to host forest therapy walks. This latter point could be applied to help alleviate some of the demands on the healthcare system by helping community members develop stronger self-care strategies for psychological wellbeing while remaining in a relatively safe and sheltered environment. Unguided nature immersion programs can be organised to encourage hospital staff, patients and community residents to immerse themselves in nature, where physiological and psychological benefits can be expected.

### 4.4. Limitations

There are some limitations to the current study. Firstly, participant recruitment did not fulfil the target as initially planned, and thus the current sample size did not fulfil the sample required as indicated by G*Power analysis. As such, findings of the current study are to be interpreted with caution and require future studies to replicate the methods and validate the results.

Second, the allocation of participants to conditions was not randomised. Thursday sessions were set to be guided conditions, and Friday sessions were set to be unguided sessions (except the two final Friday sessions). Allocation depended on whether participants signed up for the Thursday/Friday sessions, based on their own availability. While it would have been better to vary the sessions to allow random assignment, the guided session scheduling was reliant upon the availability of the certified Forest Therapy Guide (one of only two practising in Singapore at the time). Hence, with the absence of randomisation, the potential effects of lurking variables and selection bias could have influenced the results. Furthermore, some participants joined the research session alone while others joined in groups. Without randomisation, participants who signed up in groups experienced the nature immersion together with their friends, and the variation in social interaction and activity forms an important limitation as well.

Thirdly, KTPH was chosen as the venue for the research sessions to provide a more consistent and naturalistic environment, allowing for both internal and ecological validity. However, the limitations of a naturalistic setting likewise apply to the current study as well. Outside of laboratory settings, controlling for extraneous variables such as weather, humidity and surrounding noises was not possible. It is also important to note that outdoor sounds and activities that come alongside nature exposure can have a varying impact on different individuals. Sounds of cicadas in a forest may be as overwhelming as a leaf blower to some individuals. In contrast, one participant in the current study commented that the rhythmic sound from a leaf blower helped her retain focus during the water gazing segment of the guided walk sequence.

As these factors could not be held stable across different sessions and could have influenced the dependent variables such as heart rate and mood of the participants, they thus form important limitations of the experiment.

Fourth, some participants from the unguided condition returned to the meeting spot earlier than the two-hour mark and requested to end their nature immersion experience. The nature immersion duration ranged from approximately 66 min to 123 min across the 21 participants, with an average of 92 min. As participation was voluntary, it was difficult to ensure that all participants went through the same duration of nature immersion, and such variation could have influenced the data as well.

Fifth, there was little control over the activities of participants in the unguided condition. While that is the essence of the unguided condition, the variation in activity types engaged in by participants varied largely. Some participants sat under a pavilion and observed nature throughout the two hours, while others walked around the Yishun Pond Park (circumference approximately 1 km) for the entire duration. Such variation could have resulted in large differences in heart rate data, especially since it is a sensitive measure.

Lastly, small gaps in heart rate tracking were observed in the Excel datasheet downloaded off the TomTom website for most participants. There tended to be blocks of approximately five to six empty data cells, translating to missing heart rate for five to six seconds for each block. A few blocks of missing data existed per participant, and for almost all participants. This is a limitation of the heart rate tracker. The research studies supporting the efficacy of the TomTom heart rate tracker over other watch brands did not detail this issue, and the product support team were unable to provide any solution to counter the problem. Although missing data is typically a huge limitation in data analysis, the current analysis involves the heart rate averaged from more than 300,000 s. As such, the small gaps in heart rate data are not expected to pose a problem for reliability and having one participant wearing two trackers to test reliability also addresses this matter.

### 4.5. Future Research

For future research that aims to examine the efficacy of guided nature immersion programs, it is recommended to apply randomised allocation of participants to the conditions and to recruit a larger participant sample. Secondary recommendations are to offer attractive incentives to encourage participants to stay on for the full duration of unguided nature immersion and to have greater control and standardisation over the social aspect of the immersion experience and activities engaged in by participants in the unguided condition. Finally, it would be useful to conduct repeated nature immersion sessions for each participant instead of just one and to include a six-week follow-up measure of nature connectedness as well as a follow-up query as to whether participants elected to engage in their own nature immersion sessions following participation.

## 5. Conclusions

With the rise of guided nature immersion programs, it is important to test its efficacy especially since it could be a useful technique to alleviate some of the burden on the healthcare system. Findings could also potentially be used to raise awareness of environmental issues and improve engagement in pro-environmental behaviours. Besides looking at just the research literature on nature exposure, it would be useful to compare the gains from guided nature immersion programs from that of unguided nature immersion, which has yet to be investigated in research. This study aims to address the literature gap in this area. Findings revealed that, as expected, the Connectedness to Nature Scale and the Environmental Identity scale—short form showed a significant and positive correlation, indicating convergent validity of these measures. More unexpectedly, the guided nature immersion program did not offer any additional benefits compared to unguided nature immersion, but there were benefits from both in terms of nature connectedness and mood, and participants’ open-ended responses provide verbal support for the findings from the psychometric measures. Comparing within the segments of a standard sequence adopted in the practice of guided immersion programs, the third (Water gazing) and fifth (Sit spot) segments resulted in the lowest average heart rate among participants. Further studies could interrogate the effects of the various segments to better understand how each contributes to the standard sequence, providing researchers can retain reasonable consistency in the nature of these invitations.

Based on the findings, the benefits of nature exposure are once again reinforced, even in buildings and parks incorporating biophilic built design features. At KTPH where healing of physical, physiological and psychological health is of great importance, hosting of forest therapy walks (either guided/unguided) should be beneficial to the health of patients, staff and community residents. This approach is a cost-effective and simple method that can help community members develop stronger self-care strategies.

## Figures and Tables

**Table 1:** Description of participants recruited.

	Guided		Unguided		Column Totals	
	Freq.	%	Freq.	%	Freq.	%
Male	6	11.8%	6	11.8%	12	23.5%
Female	24	47.1%	15	29.4%	39	76.5%
Row Totals	30	59%	21	41%	51	
Age in years, Mean (sd)	37	(13.45)	32	(14.96)	35	(14.17)

**Table 2:** Relationship statistics for GHP with five dependent variables.

Source	*df*	Mean Square	*F*	*r*	Partial η^2^
CNSChange * GHP	1	2.498 × 10^−5^	0.001	0.982	0.00
EIDChange * GHP	1	0.010	0.051	0.822	0.001
PositiveMoodChange * GHP	1	0.024	0.079	0.779	0.002
NegativeMoodChange * GHP	1	0.127	2.48	0.122	0.050
HeartrateChange * GHP	1	0.428	0.008	0.931	0.000

Note. GHP = General Health Practice. CNS = Connectedness to Nature. EID = Environmental Identity.
